# Effect of fenugreek and flaxseed polysaccharide‐based edible coatings on the quality attributes and shelf life of apple fruit during storage

**DOI:** 10.1002/fsn3.3909

**Published:** 2023-12-27

**Authors:** Farhat Rashid, Zaheer Ahmed, Ifra Ferheen, Tahir Mehmood, Saba Liaqat, Mohammed M. Ghoneim, Afzal Rahman

**Affiliations:** ^1^ Institute of Food Science and Nutrition (IFSN) University of Sargodha Sargodha Pakistan; ^2^ Department of Nutritional Sciences & Environmental Design Allama Iqbal Open University (AIOU) Islamabad Pakistan; ^3^ School of Biosciences and Veterinary Medicine University of Camerino Camerino Italy; ^4^ Department of Food Science and Technology, Faculty of Agriculture and Environment The Islamia University of Bahawalpur Bahawalpur Pakistan; ^5^ Department of Human Nutrition and Dietetics, School of Food and Agricultural Sciences University of Management and Technology Lahore Pakistan; ^6^ Department of Pharmacy Practice, College of Pharmacy AlMaarefa University Riyadh Saudi Arabia; ^7^ Pharmacognosy and Medicinal Plants Department, Faculty of Pharmacy Al‐Azhar University Cairo Egypt; ^8^ Department of Food Technology and Rural Industries, Faculty of Agricultural Engineering and Technology Bangladesh Agricultural University Dhaka Bangladesh

**Keywords:** edible coating, food emulsions, polysaccharide, sensory analysis, shelf life

## Abstract

The present study was designed to explore the potential of fenugreek and flaxseed polysaccharide‐based edible coatings to enhance the postharvest storage life of apple fruit. The experimental plan involved the preparation of five different coating formulations, which were subsequently applied to the fruit. The coated fruit was then stored at a temperature of 25 ± 2°C for a duration of 35 days. The effects of these coatings on physicochemical and biochemical attributes (weight loss, firmness, acidity, pH, sugar content, antioxidant activity, microbial growth, and sensory properties) of coated and uncoated samples were evaluated at regular intervals: 0, 7, 14, 21, 28, and 35 days of storage. The experimental results revealed a significant difference (*p* ≤ .05) in the physicochemical parameters of uncoated and coated apple at different storage times. The coated apple fruits showed significantly (*p* ≤ .05) lower weight loss, pH, total sugars, total soluble solids, and maximum retention of ascorbic acid, firmness, acidity, and antioxidant contents, leading to enhanced organoleptic properties. The application of edible coatings extended the shelf‐life of the apples by inhibiting microbiological spoilage without substantial impact on sensory and nutritional properties. Based on these results, it is concluded that the edible coating formulation labeled *T*_1_ (containing 2.5 g fenugreek polysaccharide and 1.5 g flax polysaccharide) effectively preserved the valuable physicochemical and organoleptic characteristics of the apple fruit throughout the storage period.

## Graphical abstract

This research was aimed at determining if edible coatings based on flaxseed and fenugreek polysaccharides may extend the shelf life of apples after harvest. Without significantly altering the apples' sensory and nutritional qualities, the application of edible coatings increased their shelf life by preventing microbial deterioration. The apple's valuable physicochemical and organoleptic features were effectively retained throughout the storage period by the edible coating formulation that contained 2.5 g of fenugreek polysaccharide and 1.5 g of flax polysaccharide content.

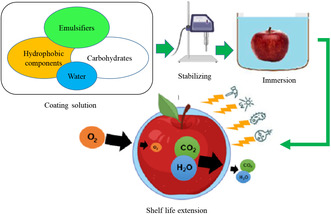

## 1 INTRODUCTION

Fruits occupy a significant position in the human diet due to their consumption being linked to various health and nutritional benefits. However, they are commodities characterized by a relatively short post‐harvest lifespan. This is primarily because they function as living tissues until they are utilized for consumption, rendering them susceptible to physiological changes that can result in substantial economic losses (Rehman et al., 2022; Zhang, Jiang, & Zhang, 2023). Throughout post‐harvest handling and storage, fruits and vegetables undergo weight loss via transpiration, a process that induces alterations in texture and surface shrinkage, thereby affecting their overall shelf stability (Ali et al., 2020; Gupta et al., 2023).

For many years, traditional synthetic waxes or chemicals were employed as post‐harvest treatments to manage deterioration and prolong the storage life of fruits (Zhang, Jiang, & Zhang, 2023). The continued utilization of these methods, however, has led to environmental and health concerns linked with chemical residues, as well as the emergence of pathogenic strains that are resistant to these treatments (Pham et al., 2023). Restrictions imposed by several countries on the use of agrochemicals, along with the growing consumer desire for high‐quality, minimally processed fresh food products, have led to the investigation of alternative preservation methods such as chemical (antioxidants, chlorination), mild heat, cold plasma, and ultrasound‐assisted techniques (Artes & Allende, 2014).

The utilization of edible coatings has emerged as an effective and eco‐friendly approach to prolonging the shelf life of fruits and vegetables while also safeguarding them from adverse environmental impacts (Gupta et al., 2023; Perveen et al., 2023). When used as coatings, these films can form semi‐permeable barriers against gases and water vapor, thus minimizing respiration and preserving the firmness of fresh produce (Pham et al., 2023).

Biopolymer‐based coatings are transparent and have a non‐greasy appearance, rendering them practical choices for improving the shelf life of fruits and vegetables due to their ability to act as water vapor barriers (Man et al., 2018; Prasad Sharma et al., 2022). Edible polymers offer numerous advantages, including easy availability, affordability, strong film‐forming potential, and the ability to create colorless and tasteless coatings that exhibit heightened oxygen barrier effectiveness (Ahmed et al., 2023; Mezhoudi et al., 2022; Pham et al., 2023). The blending of biopolymer‐based materials with other hydrophobic compounds has emerged as an economical and versatile method for developing novel materials with enhanced qualities (Ali et al., 2023; Cazón et al., 2017). Prasad Das et al. (2023) introduced a fresh and environmentally friendly approach to food systems by encapsulating essential oil within chitosan nano‐emulsions. Furthermore, Muthukumaran et al. (2022) emphasized the growing importance of polymeric biomolecule‐based nanomaterials in environmental applications.

The fenugreek (*Trigonella foenum‐graecum*) plant belongs to the Leguminosae family. The primary component found in its seed albumen is galactomannan, which is sourced from the seed endosperm (Ahmad et al., 2022; Rashid et al., 2020). When dissolved in water, galactomannan exhibits the ability to significantly increase viscosity. These characteristics render it an exceptional ingredient in comparison to other well‐known hydrocolloids, making it suitable for a range of food applications (Khorshidian et al., 2016). Furthermore, fenugreek galactomannan exhibits interface and surface tension control properties that are comparable to those of arabic gum (Sav et al., 2013). The fenugreek polysaccharide demonstrates an emulsifying potential that effectively stabilizes oil‐in‐water emulsions. This suggests that fenugreek polysaccharide possesses superior emulsifying properties in comparison to other galactomannans (Khorshidian et al., 2016). Hence, fenugreek polysaccharide can be advantageously utilized for the development of edible coatings to take advantage of the above‐mentioned benefits.

Flaxseed (*Linum usitatissimum* L.) is an annual plant classified within the Linaceae family and is cultivated for the extraction of textile fiber, seeds, and linseed oil. Flaxseed comprises a heterogeneous mixture of polysaccharides made up of xylose, galactose, ramnose, glucose, arabinose, fucose, and galacturonic acid (Zhang, Chen, et al., 2023). Polysaccharides extracted from flaxseed cake exhibit emulsifying properties closely resembling those of gum Arabic (Hamdani et al., 2019). Additionally, their water‐binding capacities are similar to those of guar gum (Mirhosseini & Amid, 2012). Hence, flaxseed polysaccharide can be advantageously utilized for the preparation of edible coatings to take advantage of the above‐mentioned benefits.

Apple (*Malus domestica* L.), a member of the Rosaceae family, stands as one of the most widely enjoyed fruits globally. Due to its exceptional nutritional profile, it holds the distinction of being the third most consumed fruit, trailing only behind citrus fruits and bananas (Bloem et al., 2023). While most of the harvested fruit is directed toward fresh consumption in markets, the necessity for storage arises to secure its availability during off‐season periods. Chemical‐free edible coatings are significant in extending the freshness and safety of food products, aligning with consumer preferences for natural and healthier food preservation techniques (Nunes et al., 2023). Given its perishable nature, the apple fruit remains vulnerable to post‐harvest losses ranging from 25 to 40% (Rab et al., 2012). The present research work was conducted to determine the effects of fenugreek and flax seed polysaccharide‐based edible coatings on the postharvest quality of apple fruit. Prior to this investigation, no studies had been conducted on this topic.

## 2 MATERIALS AND METHODS

Apples (Kala Kulu) were sourced from the Sargodha market in Pakistan for the coating process. Corn oil (Mazola) was acquired from a nearby retail grocery store. All the chemicals required for our experiments were obtained from the Pakistan Scientific Store located in Faisalabad, Pakistan.

### 2.1 Formulation and implementation of edible coating formulations

The coating formulations were prepared by adhering to the previously documented protocol (Din et al., 2015) while making slight modifications. To begin the process, we initially dispersed different concentrations of purified polysaccharide powder (obtained from fenugreek, flaxseed, and guar) in distilled water (100 mL), following the experimental treatment plan outlined in Table 1. This mixture was then heated to 50°C for a duration of 10 minutes while being continuously stirred using a magnetic stirrer. Subsequently, Tween 80 (3% w/v) and soy lecithin (1% w/v) were introduced into the solution as emulsifiers, along with a hydrophobic component (10% refined, bleached, and deodorized soybean oil). To achieve a stable emulsion, we utilized a high‐speed homogenizer operating at 1680 *g* for a duration of 5 min, effectively converting the edible coating substrate into an emulsion. The final quantity of prepared emulsion was 300 mL. We have used 250 mL of emulsion for dip treatment. After that, emulsions were cooled to room temperature and carefully stored in airtight and clean glass bottles to preserve their quality for subsequent application. For the coating process, the apples were immersed in the coating emulsions for a duration of 1 min, while a control group was treated with water (*T*_0_). Following this, the coated apples were gently twisted to remove excess coating material and left to air dry for 20 min. Once dried, the coated apples were stored in baskets under controlled conditions at 25°C ± 2, with a relative humidity ranging from 80% to 85%, for various durations of storage. Regularly, at designated time intervals (0, 7, 14, 21, 28, and 35 days), the fruits were extracted from storage for a series of evaluations. These evaluations encompassed physicochemical assessments, microbial analysis, and sensory evaluations, comparing the coated and uncoated samples to monitor their quality throughout the storage period.

**TABLE 1:** Treatment plan of polysaccharide‐based coating formulation.

Treatments	Quantity (g)
*T*_0_	Control (untreated)
*T*_1_	Fenugreek (2.5 g) + Flaxseed (1.5 g)
*T*_2_	Fenugreek (4 g)
*T*_3_	Flaxseed (4 g)
*T*_4_	Guar (4 g)

### 2.2 Physiochemical analyses

The assessment of fruit weight reduction, pH levels, acidity, total soluble solids (TSS), and the content of ascorbic acid was conducted using the previously established methodology (AOAC, 2006). The firmness of the fruit was determined using a penetrometer (T.R. Turoni Co. Snc., Italy) and quantified in kilogram‐force. The determination of reducing and total sugar content in apple juice was carried out through the method of Lane and Eynon, as reported by Ranganna (1986).

### 2.3 Antioxidant activity

The assessment of antioxidant capacity was conducted by evaluating its ability to scavenge stable DPPH free radicals, following the methodology originally proposed by Yen and Chen (1995).

### 2.4 Microbiological analyses

The microbial analysis, including total plate count (TPC) and mold and yeast count, was conducted following the procedure outlined by Siroli et al. (2015). In brief, samples (10 g) were submerged in a saline solution (90 mL), agitated for 2 min at room temperature, and subsequently diluted using a sterile saline solution. For the TPC, the samples were incubated for 48 h at 37°C on plate count agar, while for yeast and molds, incubation was carried out at 30°C on potato dextrose agar for 48 h.

### 2.5 Sensory evaluation

A group of ten partially trained evaluators conducted an assessment of sensory evaluation, including taste, flavor, color, and overall satisfaction for both coated and uncoated samples. The samples were evaluated on a 9‐point hedonic scale.

### 2.6 Statistical analysis

The experimental findings were subjected to analysis of variance (ANOVA) to determine if there were any statistically significant differences among the variables by adding the *F*‐value at .001, .01, and .05 probability. In the second phase, the results were compared by applying the least significant difference (LSD) test using analysis of variance at the 5% level of significance.

## 3 RESULTS AND DISCUSSION

In our prior publication, we reported the purification and characterization of polysaccharides derived from fenugreek and flaxseeds for the purpose of creating edible coatings (Rashid et al., 2018, 2019).

### 3.1 Weight loss (%)

**FIGURE 1:**
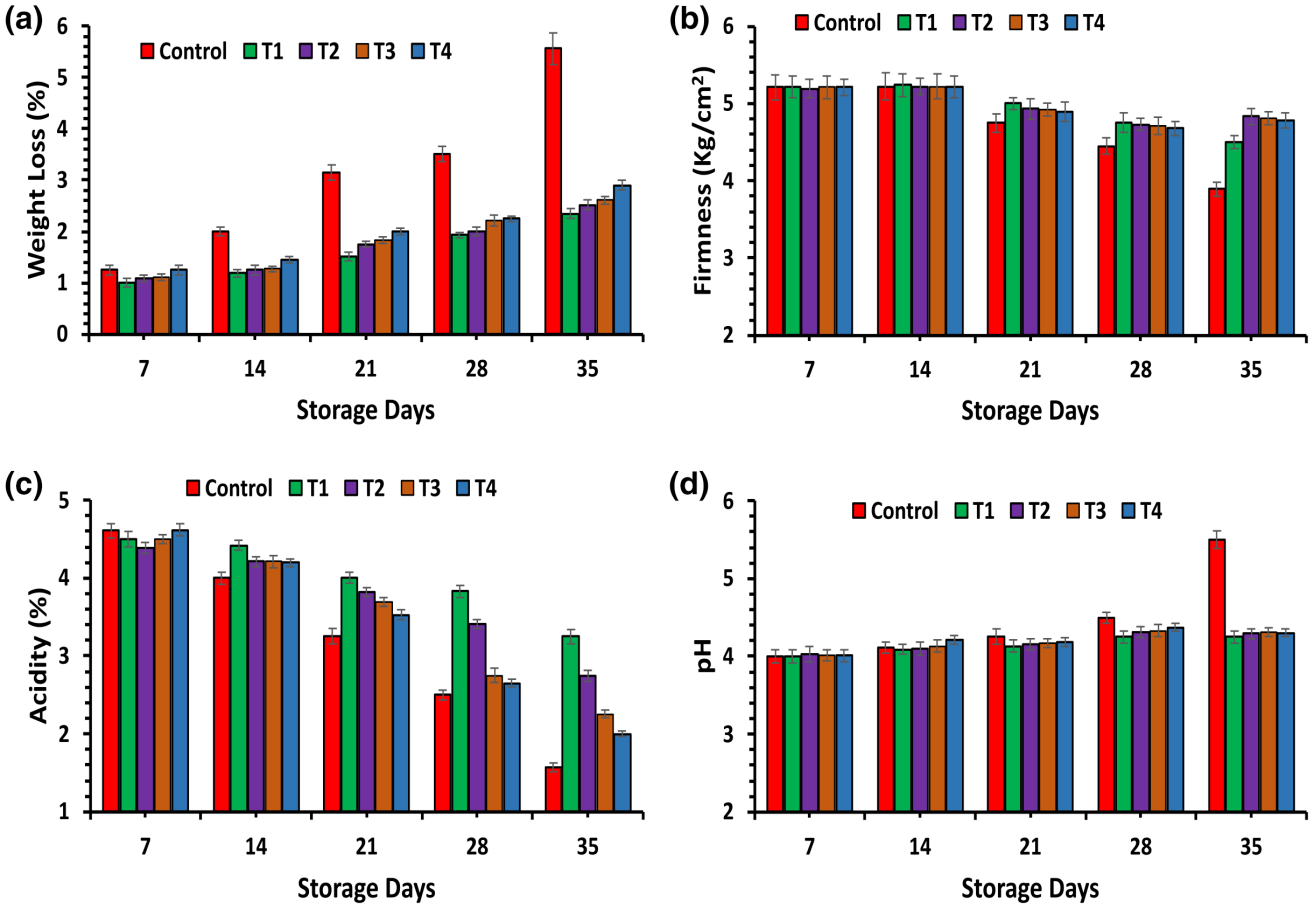
Effect of polysaccharide‐based edible coating treatments on Weight Loss (a), Firmness (b), Acidity (c), and pH (d) on apple fruit stored at room temperature (25 ± 2°C).

The presence of water in fruits can trigger various reactions, including browning, increased microbial growth, alterations in texture, vitamin degradation, and heightened enzyme activity. These factors collectively render fruits more susceptible to deterioration (Jideani et al., 2017). The ANOVA results revealed a significant impact (*p* ≤ .05) of various treatments, duration of storage, and interaction of treatments and storage on the weight loss observed in apple fruits, as illustrated in Figure 1a. As the storage duration progressed, a noteworthy distinction (*p* ≤ .05) became evident between coated and uncoated fruits. When comparing the means of different treatments, it was observed that the weight loss had consistently increased over time for both coated and uncoated fruits during storage. In a statistical context, it was found that uncoated apples (*T*_0_) experienced the highest weight loss, reaching 5.56 ± 0.3% after 6 weeks of storage. In contrast, all the coated fruits in this study exhibited significantly reduced weight loss compared to the uncoated ones. Minimum weight loss was observed in *T*_1_, with a value of 2.35 ± 0.09%, followed by *T*_2_ (2.52 ± 0.10%), *T*_3_ (2.61 ± 0.07%), and *T*_4_ (2.90 ± 0.10%) for the coated apple fruits, as depicted in Figure 1a.

Fresh fruits lose weight during storage due to respiration and the evaporation of moisture (Ali et al., 2020). The weight loss depends on different factors such as storage conditions, temperature, and applications of different coatings (Pham et al., 2023; Rehman et al., 2022). Jahanshahi et al. (2018) demonstrated the effectiveness of applying a tragacanth gum‐based coating to preserve the water content in two cultivars of apple during storage. These types of gums act like a protective layer on the fruit's surface, preventing water from evaporating and reducing the weight loss of fruits (Riederer et al., 2015). Moreover, a variety of edible coatings composed of polysaccharides, either in isolation or in combination, have been utilized to reduce weight loss in various fruits. For instance, sugar apples were coated with gum Arabic and carboxymethyl cellulose (Man et al., 2018), and fresh‐cut apples were treated with sodium alginate and pectin (Guerreiro et al., 2017).

### 3.2 Firmness (kg/cm^2^)

Firmness is a pivotal factor in determining the edible quality, consumer satisfaction, and market worth of both fruits and vegetables. The ANOVA results revealed a significant impact (*p* ≤ .05) of various treatments, duration of storage, and the interaction of treatment and storage on the firmness of apple fruits, as depicted in Figure 1b. The results of this experiment depicted a consistent reduction in fruit firmness throughout the storage period for all treatments. This decline is particularly noticeable in uncoated fruits. However, a combination of fenugreek and flaxseed polysaccharide in treatment *T*_1_ maintained significantly higher firmness (4.50 ± 0.09 kg/cm^2^), followed by *T*_2_ (4.83 ± 0.11 kg/cm^2^), *T*_3_ (4.81 ± 0.08 kg/cm^2^), and *T*_4_ (4.78 ± 0.10 kg/cm^2^), respectively, after the storage of 6 weeks, as depicted in Figure 1b. The retention of greater fruit firmness in this treatment may be due to the use of composite coatings (fenugreek and flaxseed polysaccharide‐based edible coatings) that maintain the integrity of the cell wall and reduce oxidative stress, which result in firmer apple fruit (Shakir et al., 2022). Edible coatings might have hindered the action of enzymes that break down pectin by slowing metabolic processes, ultimately resulting in firmer apple fruits (Rab et al., 2012). These results are consistent with prior research by Jahanshahi et al. (2018), which reported that coatings of tragacanth gum effectively maintained the firmness in two cultivars of apple, specifically Golden Delicious and Red Delicious, during storage compared to apple fruits without coating.

### 3.3 Acidity (%)

The outcomes of the ANOVA demonstrated a notably significant impact (*p* ≤ .05) of various treatments, time intervals, and the interaction of storage and treatment on the acidity levels of apple fruits, as depicted in Figure 1c. The findings indicated that titratable acidity values decreased over the course of storage for both coated and uncoated apple fruits. Coatings could potentially reduce the respiration rate of the fruit, which may slow down metabolic processes, including acid breakdown by reducing gas exchange to the external environment (Ghidelli & Pérez‐Gago, 2018). However, when comparing treatment means, it became evident that all coating treatments maintained varied titratable acidity levels compared to the uncoated fruit. In all coating treatments, it was observed that fruit in treatment *T*_1_ exhibited a smaller reduction in titratable acidity (3.25 ± 0.09%), followed by *T*_2_ (2.75 ± 0.07%), *T*_3_ (2.25 ± 0.05%), and *T*_4_ (2.00 ± 0.06%) after 35 days of storage. In contrast, uncoated fruit in *T*_0_ (1.57 ± 0.05%) experienced a higher reduction in titratable acidity after storage, as depicted in Figure 1c.

Our findings contrast with the research findings of Jahanshahi et al. (2018), which demonstrated that tragacanth gum‐coated apple fruits exhibited a statistically significant increase in titratable acidity during storage. In accordance with prior research conducted by Ullah et al. (2017), the titratable acidity gradually declined in fruits treated with elevated concentrations of CMC + carrageenan, chitosan, and gum Arabic. This decline was attributed to the prevention of organic acid degradation in fruits during storage. Additionally, coatings increase the retention of titratable acidity in fruits by slowing down metabolic reactions and respiration (Shah et al., 2015).

### 3.4 pH

The ANOVA has unveiled a notably significant influence (*P* ≤ .05) of various treatments, durations of storage, and their interplay on the pH levels of coated fruits throughout the storage period, as illustrated in Figure 1d. In our present study, when comparing the treatment means, it became evident that the pH levels increased progressively as the storage duration advanced. The highest pH value was observed in uncoated *T*_0_ (5.5 ± 0.12), followed by *T*_3_ (4.31 ± 0.08), while a declining trend in pH values was observed in *T*_1_ (4.25 ± 0.08), *T*_2_ (4.29 ± 0.07), and *T*_4_ (4.30 ± 0.06), respectively, at the conclusion of the storage period, as depicted in Figure 1d. These findings indicate that the treated fruits were able to preserve a consistent level of pH in contrast to the control fruits, presumably due to the coating's ability to slow down metabolic reactions. Menezes and Athmaselvi (2016) similarly documented that the application of polysaccharide‐based edible coatings has the capacity to retard alterations in fruit pH. Bilawal et al. (2017) and Ullah et al. (2017) obtained analogous findings in their research, suggesting that the decline in pH may be linked to physiological, structural, and biochemical modifications occurring during respiration. On the flip side, the increase in pH may be linked to the buildup of dry matter content and the depolymerization of polysaccharides as storage progresses.

### 3.5 Total soluble solids (°brix)

**FIGURE 2:**
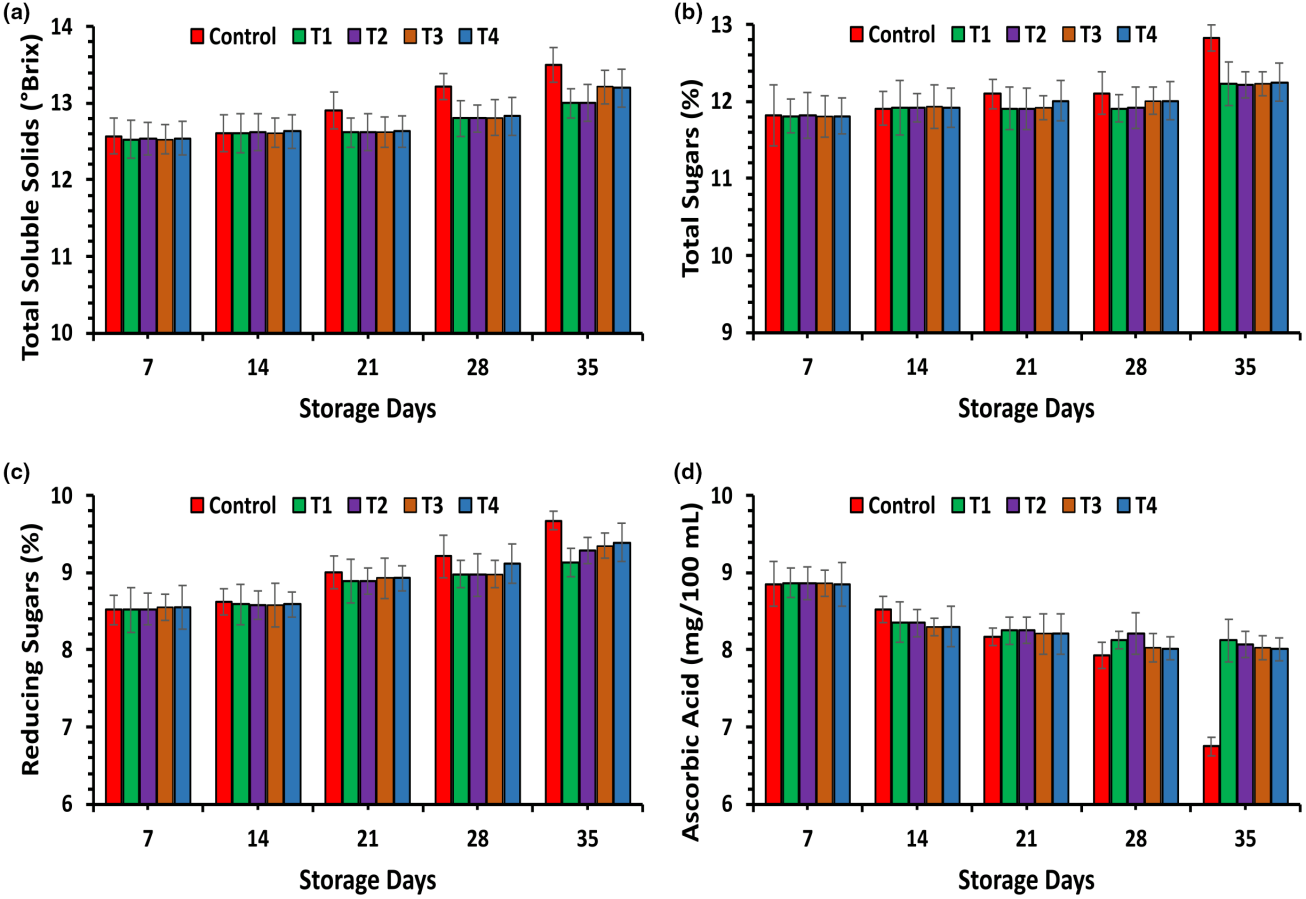
Effect of polysaccharide‐based edible coating treatments on Total soluble solids (a), Total sugars (b), Reducing sugars (c), and Ascorbic acid (d) on apple fruit stored at room temperature (25 ± 2°C).

The ANOVA revealed a significant impact (*p* ≤ .05) of various treatments, time intervals, and their interactions on the total soluble solids (TSS) of apple fruits, as depicted in Figure 2a. In this study, the TSS of apple fruits was notably influenced by the coating treatments, the duration of storage, and their interplay, as demonstrated in Figure 2a. As the storage period advanced, a noteworthy distinction (*p* ≤ .05) emerged among the means of treatment for both uncoated and coated fruits. Both the apple fruits with edible coatings and the control group exhibited an increase in TSS. However, the coating treatments significantly slowed down the rate of TSS increase in apple fruits throughout the storage duration. After six‐week storage period, uncoated *T*_0_ samples (13.50 ± 0.22 °Brix) displayed the maximum TSS, possibly attributable to the breakdown of complex sugars into simpler sugars during storage (Hesami et al., 2021).

Similarly, a notably lower TSS value was noted in treatment *T*_1_ (13.00 ± 0.19 °Brix), followed by *T*_2_ (13.1 ± 0.24 °Brix), *T*_3_ (13.21 ± 0.22 °Brix), and *T*_4_ (13.28 ± 0.09 °Brix), respectively, as depicted in Figure 2a. Our findings aligned with the results obtained by Wijewardane and Guleria (2009), who also noted a gradual increase in TSS values of apples coated with corn starch, rice, and potato when compared to uncoated fruits during storage. Similarly, patterns were noted in the storage of carrageenan and CMC‐coated papaya (Vyas et al., 2014) and gum arabic and soya bean‐coated anna apple (El‐Anany et al., 2009).

### 3.6 Total sugar (%)

The ANOVA analysis indicated a significant impact (*p* ≤ .05) of various treatments, durations of storage, and their interactions on the total sugar content of apple fruits throughout the storage period, as depicted in Figure 2b. These results indicate that, irrespective of the coating treatments applied, there was a consistent rise in the total sugar content as the storage duration progressed. However, it is noteworthy that throughout the storage period, all treatments utilizing flaxseed and fenugreek polysaccharide coatings distinctly slowed down the sugar accumulation process and maintained a lower total sugar compared to the control sample. The highest sugar (12.83 ± 0.17%) was observed in *T*_0_, while the lowest (12.23 ± 0.28%) was recorded in *T*_1_. Additionally, at the conclusion of the storage period, *T*_2_ (12.22 ± 0.17%) and *T*_3_ (12.19 ± 0.16%) displayed a lower concentration of sugar, followed by *T*_4_ (12.25 ± 0.25%), in comparison to the uncoated fruits, as depicted in Figure 2b. Hence, the data clearly indicates that the control group had significantly higher total sugar values, whereas the treated fruits displayed the smallest increment in total sugar content throughout their storage period.

A comparable study was conducted by Zapata et al. (2008), who noted that coating tomatoes with zein and alginate exhibited lower organic acid concentrations and sugar levels when compared to control samples. Joshi et al. (2017) similarly observed that the utilization of a gum ghatti‐based coating emulsion markedly reduced the sugar content in coated papayas by inhibiting metabolic processes such as the breakdown of starch and the effects of various enzymes.

### 3.7 Reducing sugar (%)

The ANOVA analysis demonstrated a significant impact (*p* ≤ .05) of various treatments, durations of storage, and their interplay on the reducing sugar content of apples coated with edibles, as illustrated in Figure 2c. An analysis of the treatment means showed that the proportion of reducing sugar rose with the prolongation of the storage duration. The results revealed that the highest percentage (9.67 ± 0.12%) of reducing sugar was observed in *T*_0_, while the lowest percentage (9.13 ± 0.18%) was recorded in *T*_1_, followed by *T*_2_ (9.29 ± 0.17%) after 6 weeks of storage. Additionally, there was no significant difference observed between treatment *T*_3_ (9.35 ± 0.16%) and *T*_4_ (9.39 ± 0.25%), as depicted in Figure 2c. A corresponding discovery was validated through experimental evidence by Sariful et al. (2001), who demonstrated a continuous rise in both reducing and total sugar content during the storage of bananas. Likewise, Jan and Rab (2012) reported a similar outcome for apple cultivars throughout the storage period.

### 3.8 Ascorbic acid (mg/100 mL) content

The ANOVA analysis demonstrated significant results (*p* ≤ .05) for the different treatments, durations of storage, and their interactions, as depicted in Figure 2d. The results obtained in this study indicate a consistent decrease in the ascorbic acid content across all treatments, likely due to its oxidation. When comparing the means of treatments, the highest ascorbic acid content (8.12 ± 0.28 mg/100 mL) was observed in *T*_1_, followed by *T*_2_ (8.07 ± 0.17 mg/100 mL), *T*_3_ (8.00 ± 0.16 mg/100 mL), and *T*_4_ (7.56 ± 0.15 mg/100 mL), respectively. Conversely, the lowest ascorbic acid content (6.75 ± 0.12 mg/100 mL) was observed in the control group, *T*_0_, after storage, as depicted in Figure 2d. In this study, the higher ascorbic acid concentration in the coated samples compared to the control may be attributed to the enhanced oxygen barrier properties of the coating material, which results in a reduced rate of respiration.

The results of our present study closely align with the findings of Ayranci and Tunc (2003), who noted that the densely layered network structure in edible coatings leads to decreased oxygen permeability in the coated product. This reduction in oxygen availability helps to prevent the oxidation of ascorbic acid. A similar trend of reduced ascorbic acid loss was observed in lychee fruits, carambola, and green grapes when composite coatings consisting of xanthan gum, alginate, olive oil, and chitosan were employed (Baraiya et al., 2015, 2018).

### 3.9 Antioxidant capacity (%)

**FIGURE 3:**
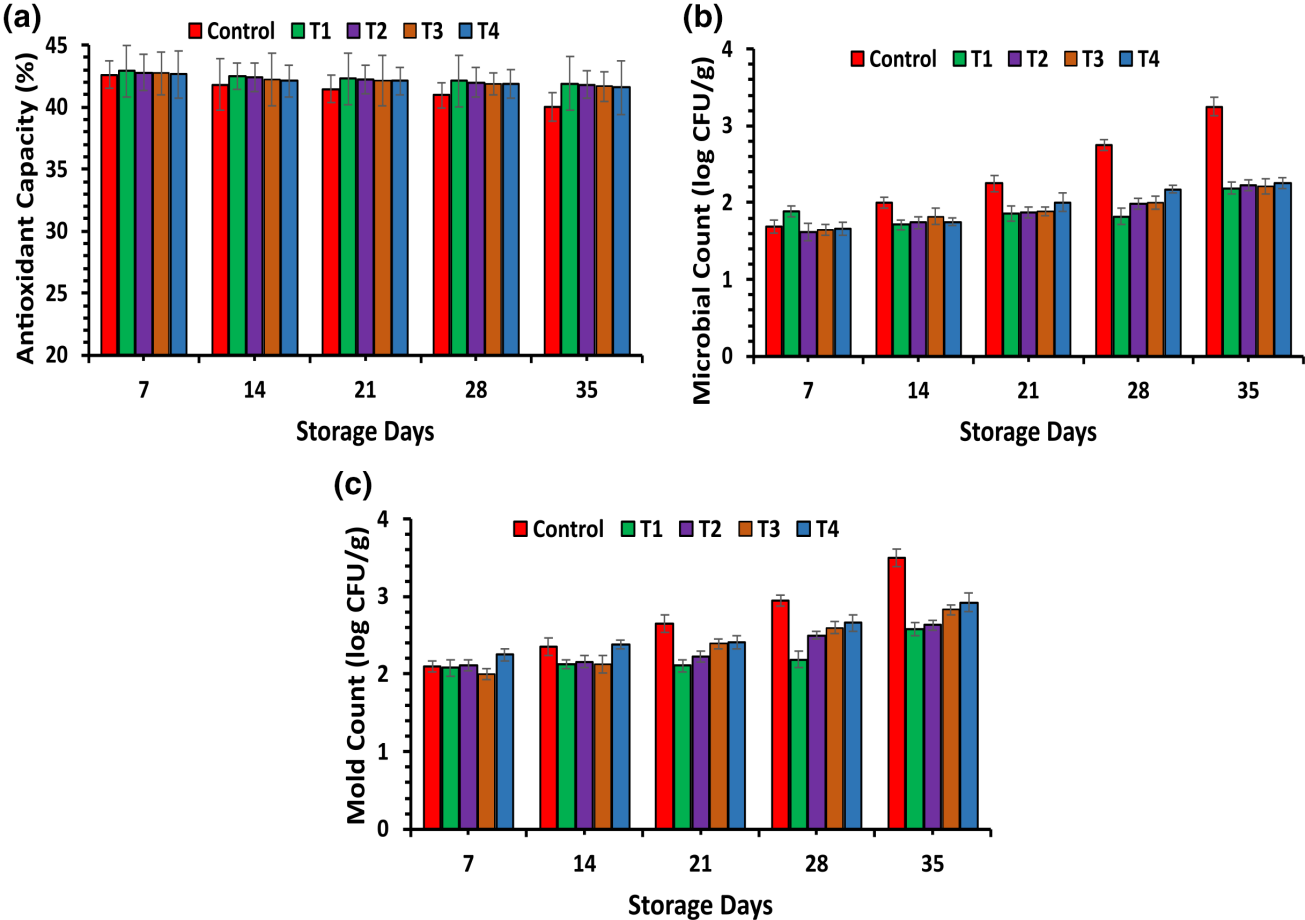
Effect of polysaccharide‐based edible coating treatments on Antioxidant capacity (a), Microbial count (b), and Mold count (c) on apple fruit stored at room temperature (25 ± 2°C).

The ANOVA results demonstrated significant effects (*p* ≤ .05) concerning various treatments, storage periods, and their interactions, as depicted in Figure 3a. The outcomes of this study indicate a decline in antioxidant activity across all tested fruits during the storage period. When comparing the means of treatments, it was evident that the highest antioxidant activity was observed in *T*_1_ (41.91 ± 2.20%), followed by *T*_2_ (41.81 ± 1.10%), *T*_3_ (41.69 ± 1.19%), and *T*_4_ (41.58 ± 2.16%), while the lowest level was noted in *T*_0_ (40.01 ± 1.14%), as depicted in Figure 3a. The total antioxidant activity was reduced in the uncoated fruit but remained comparatively stable in the coated fruit over the six‐week storage period. This stability confirms that the combination of flaxseed and fenugreek gums as a coating on fruits effectively controls oxidative stress within the coated fruits (Tahir et al., 2019). Our findings align with the results reported by Correa‐Betanzo et al. (2011), who noted that the use of edible coatings had a stabilizing effect on antioxidant activity compared to control fruit. Similarly, Saba and Sogvar (2016) also observed a similar trend, where the combined effect of CMC‐based coatings significantly reduced antioxidant capacity during storage in uncoated fruits, while CMC‐coated fruits maintained their antioxidant capacity at levels similar to the first day.

### 3.10 Microbial analysis

#### 3.10.1 Total plate count (log CFU/g)

The ANOVA analysis demonstrated significant results (*p* ≤ .05) with respect to different treatments, durations of storage, and their interaction concerning the total bacterial count of fruit samples, as illustrated in Figure 3b. The experimental results revealed that the total viable counts steadily rose as the storage period advanced for all treatments, irrespective of whether they were coated or uncoated. Throughout the storage period, the microbial count in all the coated apple samples consistently remained lower than that in the uncoated sample, even at the conclusion of the storage duration. Among the treatments, *T*_1_ (2.19 ± 0.08 log CFU/g) with a coating of flaxseed and fenugreek polysaccharide exhibited the most effective antimicrobial properties, followed by *T*_3_ (2.21 ± 0.10 log CFU/g), *T*_2_ (2.23 ± 0.07 log CFU/g), and *T*_4_ (2.26 ± 0.07 log CFU/g), as compared to the control sample, which had a microbial count of (3.25 ± 0.12 log CFU/g) at the end of 6‐week storage, as shown in Figure 3b. The synergistic effect of a mixture of flaxseed and fenugreek coating was observed due to the modification of the both physical and rheological characteristics of the composite coating (Shakir et al., 2022).

#### 3.10.2 Mold and yeast counts

The ANOVA analysis revealed significant differences (*p* ≤ .05) in the CFU/g values for yeast and molds in various treatments, durations of storage, and their interactions, as depicted in Figure 3c. The experimental findings indicated that the mold and yeast counts were recorded at 2.08 ± 0.11, 2.10 ± 0.07, 2.00 ± 0.07, and 2.25 ± 0.09 log CFU/g for different treatments of coated apple fruit such as *T*_1_, *T*_2_, *T*_3_, and *T*_4_, respectively. In contrast, the control sample (uncoated) had a count of 2.11 ± 0.07 log CFU/g, as shown in Figure 3c. However, these counts exhibited a linear increase as the storage period advanced for all tested samples, with a notable difference observed in the control sample compared to the other apple samples. Importantly, even after 6 weeks of storage, the yeast and mold counts in the coated apple fruit remained lower than the control sample. At the end of the storage study, the yeast and mold counts for the treated apples were recorded as 2.58 ± 0.08, 2.63 ± 0.07, 2.83 ± 0.06, and 2.92 ± 0.12 log CFU/g for treatments *T*_1_, *T*_2_, *T*_3_, and *T*_4_, respectively. In contrast, the control sample (*T*_0_) exhibited a mold and yeast count of 3.50 ± 0.12 log CFU/g. The inhibition of microbial growth observed in this study aligns with the antimicrobial properties exhibited by different gums, such as CMC and sodium alginate, in maintaining the quality and shelf stability of persimmon fruits (Hegazy, 2017). Additionally, similar antimicrobial effects were reported for alginate coatings applied to apple slices (Chauhan et al., 2011; Moreira et al., 2015) and kiwifruit slices (Benítez et al., 2015), resulting in a reduction in yeast, mold, and mesophilic aerobic bacteria growth during storage.

### 3.11 Sensory evaluation

**FIGURE 4:**
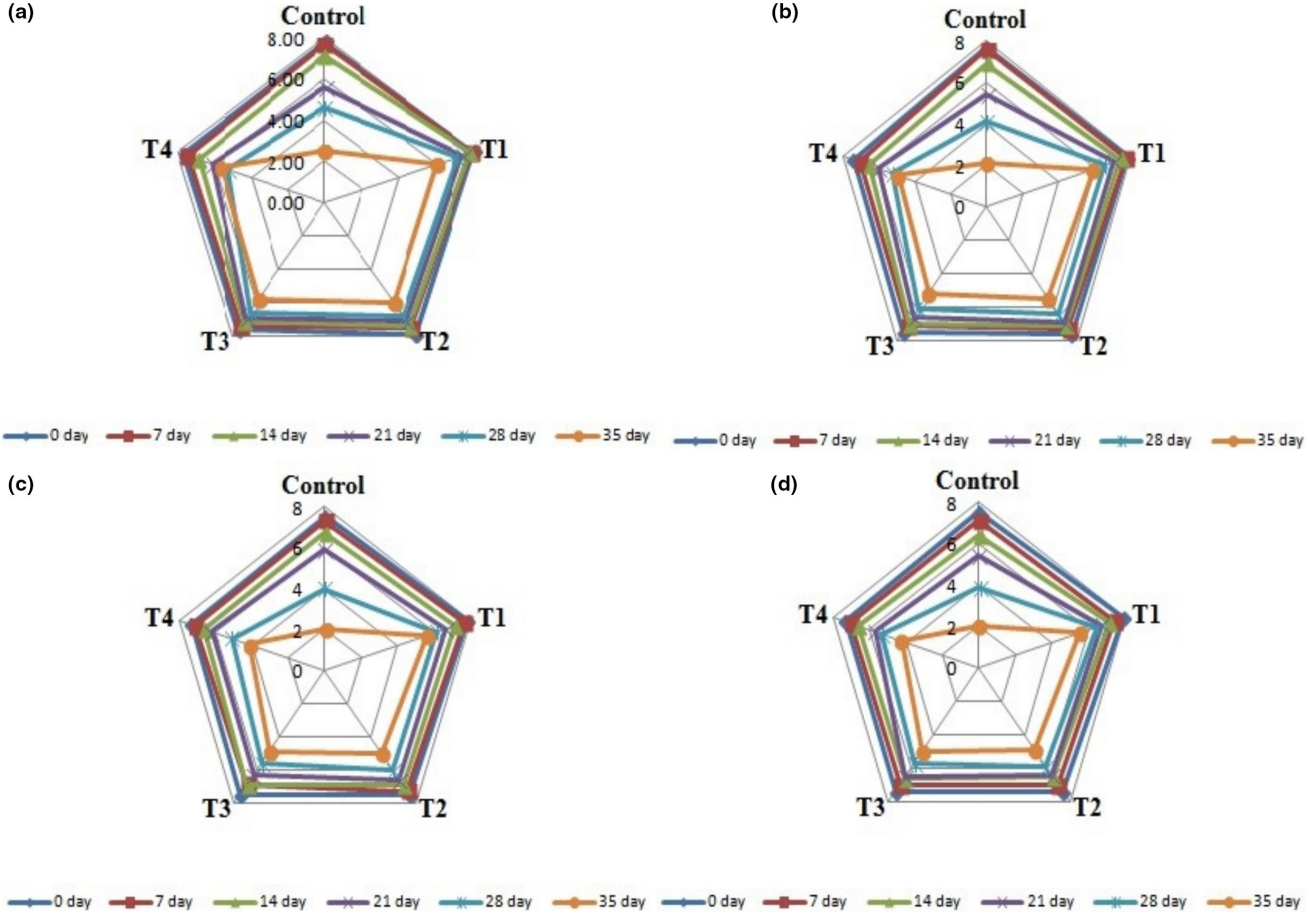
Effect of polysaccharide‐based edible coating treatments on organoleptic properties of apple fruit stored at room temperature (25 ± 2°C) on different storage days (0, 07, 14, 21, 28, and 35 days), i.e., Color (a), Flavor (b), Taste (c), and Overall acceptability (d).

The ANOVA analysis yielded a significant impact (*p* ≤ .05) concerning the effect of treatments, time period, and their interaction regarding the color, flavor, taste, and overall acceptability of edible coated fruits, as illustrated in Figure 4a–d. When comparing the means of treatments, it was evident that the highest scores were achieved by treatment *T*_1_, followed by *T*_2_, *T*_3_, and *T*_4_, respectively, on the 28th day of storage, while the control sample received the lowest score, as shown in Figure 4. The findings suggested that sensory acceptability progressively declined with the passage of time across all treatment groups. However, polysaccharide‐based coatings were able to significantly (*p* ≤ .05) maintain their organoleptic properties during storage compared to the control sample. Our results are consistent with studies conducted by Al‐Juhaimi et al. (2012), which demonstrated that the coatings of Arabic gum effectively extend the storage life and preserve the sensory characteristics (green color and brightness) of cucumber fruit during storage at 10 and 25°C. Similar findings were reported by Tahir et al. (2018), who highlighted the positive impact of gum Arabic on postharvest shelf life and the sensory quality of strawberries during storage. Additionally, Ergun and Satici (2012) also noted that apples coated with aloe vera gel retained better quality characteristics and appearance compared to control fruits during storage.

## 4 CONCLUSION

The results from the current study demonstrated that the application of edible coatings in combination with fenugreek (2.5 g) and flaxseed (1.5 g) polysaccharides can be used as an effective treatment. This synergistic treatment (fenugreek and flaxseed gum coating mixture) successfully reduces microbial contamination while preserving the quality attributes of fruits without significantly altering their nutritional value when stored at room temperature (25 ± 2°C). Consequently, the study concludes that edible coatings, when used synergistically, offer a successful strategy for reducing postharvest losses and enhancing the shelf stability of apple fruits during storage.

## AUTHOR CONTRIBUTIONS

**Farhat Rashid:** Conceptualization (equal); formal analysis (equal); methodology (equal); writing – original draft (equal); writing – review and editing (equal). **Zaheer Ahmed:** Conceptualization (equal); formal analysis (equal); methodology (equal); supervision (equal); validation (equal); writing – original draft (equal). **Ifra Ferheen:** Conceptualization (equal); formal analysis (equal); methodology (equal); writing – original draft (equal). **Tahir Mehmood:** Conceptualization (equal); formal analysis (equal); methodology (equal); supervision (equal); writing – original draft (equal); writing – review and editing (equal). **Saba Liaqat:** Conceptualization (equal); formal analysis (equal); methodology (equal); writing – original draft (equal); writing – review and editing (equal). **Mohammed M. Ghoneim:** Formal analysis (equal); supervision (equal); writing – original draft (equal); writing – review and editing (equal). **Afzal Rahman:** Conceptualization (equal); formal analysis (equal); methodology (equal); writing – original draft (equal).

## CONFLICT OF INTEREST STATEMENT

Authors have no conflict of interest to declare.

## Data Availability

The data that support the findings of this study are available on request from the corresponding author.
